# Environmental stressors influencing hormones and systems physiology in cattle

**DOI:** 10.1186/1477-7827-12-58

**Published:** 2014-07-04

**Authors:** Toree L Bova, Ludovica Chiavaccini, Garrett F Cline, Caitlin G Hart, Kelli Matheny, Ashleigh M Muth, Benjamin E Voelz, Darrel Kesler, Erdoğan Memili

**Affiliations:** 1 Department of Animal and Dairy Sciences, College of Agriculture and Life Sciences, Mississippi State University, Mississippi State, MS 39762, USA; 2 Department of Clinical Sciences, College of Veterinary Medicine, Mississippi State University, Mississippi State, MS 39762, USA; 3 Department of Animal Sciences, College of Agricultural, Consumer and Environmental Sciences, University of Illinois at Urbana Champaign, Urbana, IL 61801, USA

**Keywords:** Systems physiology, Environmental stressors, Endocrine system

## Abstract

Environmental stressors undoubtedly influence organismal biology, specifically the endocrine system that, in turn, impact cattle at the systems physiology level. Despite the significant advances in understanding the genetic determinants of the ideal dairy or beef cow, there is a grave lack of understanding of the systems physiology and effects of the environmental stressors that interfere with the endocrine system. This is a major problem because the lack of such knowledge is preventing advances in understanding gene-environment interactions and developing science-based solutions to these challenges. In this review, we synthesize the current knowledge on the nature of the major environmental stressors, such as climate (heat, cold, wind, and humidity), nutrition (feeds, feeding systems, and endocrine disruptors) and management (housing density and conditions, transportation, weaning practices). We summarize the impact of each one of these factors on cattle at the systems level, and provide solutions for the challenges.

## Background

Systems physiology is a scientific discipline combining theoretical, computational, and experimental studies to increase our understanding of the physiology of living creatures [1]. Understanding organism biology requires a systems physiology approach to dissect molecular and cellular mechanisms that regulate phenotypes, development and disease of cattle. Homeostasis is essential for cattle to achieve and sustain health and, indirectly, food productions and requires hormones, powerful substances secreted by various organs in the body responsible for stimulating a cell-specific response. Stress is defined as a condition in an animal that results from the action of one or more stressors of either external or internal origin. Adjusting to stress induces a wide range of behavioral and physiological responses including endocrine changes in the hypothalamus-pituitary-adrenal (HPA) axis thus releasing corticosteroids and aldosterone [2]. The overall effects on the animal are multifaceted, so different physiological outputs must be studied in order to understand the global effects of stimuli on animals and how these influence animal health, production, and quality food products.

**Figure 1:**
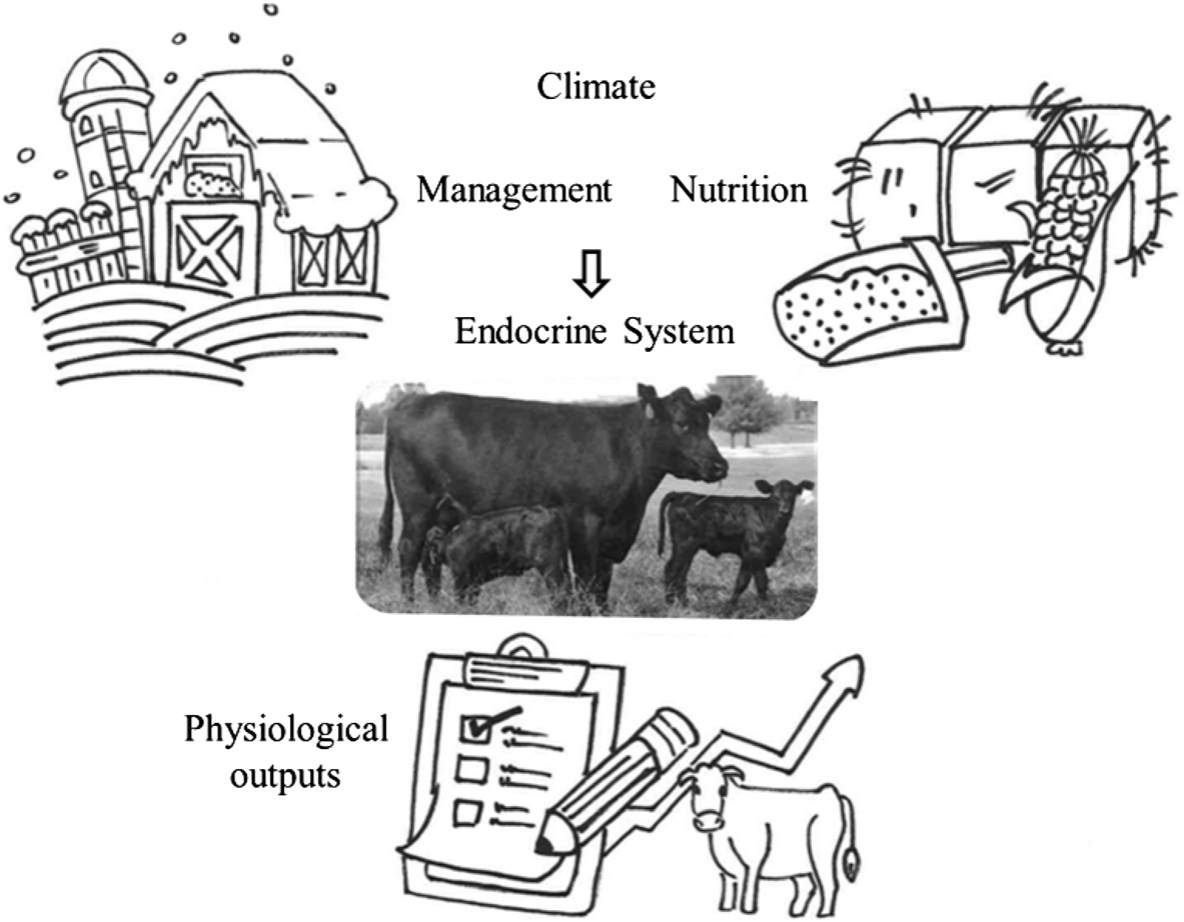
**Systemic effects of environmental factors through endocrine system.** Environmental factors such as climate, nutrition and management have an effect on diverse hormones which in turn impact systems physiology within the animal. Physiological outputs from each system can be measured to determine the mechanisms regulating animal reproduction, growth and development, health, production and product quality.

The environmental factors of climate, nutrition, and management are considered major stressors on animal health and production. Those external factors or stimuli are transduced by different receptors and may result in epigenetic changes in the absence of any changes in gene sequence in cattle. Although it is well known that phenotypes are influenced by both the genotype and the environment, major gaps still exist in the current understanding of mechanisms by which environmental factors modulate phenotype, development and disease within an organism. For example, the level of feeding of sires was shown to affect milk production in daughters [3], and dietary treatments were shown to hasten the onset of puberty and altered gene expression in the arcuate nucleus of beef heifers [4]. Also, it is known that season can have major impacts on the reproduction, lactation and growth of cattle [5]. However, environmental effects on the genome and epigenome must be investigated further before they may be used to predict performance and improve selection for superior animals.The objective of this review is to synthesize several key environmental factors that influence the endocrine system that subsequently affects cattle at the systems physiology level. Disruptions in physiology at the systemic levels can lead to diminished returns from cattle production in a multitude of ways. This review summarizes the current knowledge of managerial, climatic and nutritional stressors, and in doing so, sheds light on areas that require further study; including the mechanisms that allow stressors to disrupt endocrine function (Figure 1). This review is helpful to not only professionals within the livestock production industry such as extension agents, but to provide an overview of past research and a direction for future research to scientists in a variety of fields.

### Environmental factors influencing endocrine systems and cattle physiology

#### Management, hormones, and physiological outputs

In order to maximize production, modern farming practices call for intensive management of livestock. Management practices such as handling, weaning, housing conditions and transportation are essential to the well-being and production efficiency of cattle but are also major stressors which can cause a decrease in their productivity. Increased cortisol concentrations have been shown to increase anxiety-related activity [6,7], disease susceptibility [2] as well as decreased reproductive performance [8,9] of cows and for this reason cortisol is often used as a measure of stress. The effects of stress on productivity have been widely studied; however, the mechanisms of action are poorly understood.

Stress caused by housing can result from a number of identifiable housing conditions or structure including ventilation, cooling, flooring, animal density, and regrouping of the animals. Insufficient ventilation and cooling systems can aggravate stress induced by exposure to extreme heat or cold. The impact of extreme heat or cold can vary widely depending on the stage of life and reproductive status of animals. Housing bulls in high density situations (1.2 m^2^ per animal) results in an acute rise in plasma cortisol concentration that is detrimental to growth. Bulls housed at 4.2 m^2^ per animal had depressed interferon-gamma production when compared to those housed at 2.7 and 1.2 m^2^ per animal [10]. Interferon-gamma is a mediator of immunological and pathological response to stress [11]. Therefore, it can be concluded, that animals housed in lower density situations are less stressed than those in higher densities. It has been demonstrated that social stress due to hierarchy activates the adrenal-cortical axis, increases cortisol and catecholamine production and, in the long term, can affect the cardiovascular function, fertility, immunosuppression and neurologic dysfunction [12]. Cattle regrouped into cohorts of similar characteristics such as age or reproductive status require re-establishment of the social dominance hierarchy, which has been shown to affect time spent at the feeder which resulted in decreased milk production in lower-ranking females [13].

Weaning is a natural process and can induce stress in both the calf and the dam. Norepinephrine and epinephrine, major neurotransmitters of the sympathetic autonomic nervous system, are both affected by stress. Norepinephrine concentrations increase in calves while epinephrine concentrations increase in both calves and dams during separation, but decrease again after reunion [14]. However, weaning calves at day 89 compared to day 300 has been shown to increase the tolerance of these calves to transport and feedlot entry stress. Acute-phase proteins, haptoglobin and ceruloplasmin, as part of the innate immune system, were measured as an estimate of stress. While haptoglobin concentrations were increased in both groups of calves, calves weaned at day 89 showed lesser ceruloplasmin concentrations when compared to calves weaned at day 300 as well as improved feed to gain ratios [15]. Transportation is a managerial necessity that can cause stress on cattle. Adrenocorticotropin (ACTH), a hormone secreted by the anterior pituitary in response to stressful stimuli such as transportation, causes the release of glucocorticoids from the adrenal glands. This results in immunosuppression and changes in the regulation of glucose homeostasis. It has also been found that peripheral lymphocytes secrete ACTH during times of stress. Lymphocytic ACTH secretion was significantly increased in cattle transported for 14 hours and remained at elevated when animals rested on the transportation trailer. However, lymphocytic ACTH levels returned to pre-transport levels when the cattle were offloaded and rested in stalls [16].

In *Bos taurus* beef females, acclimation to human handling after weaning actually expedites reproductive development in replacement heifers [8]. Repeated regrouping and repenning showed no sustained detrimental effect on production or immune measurements in steers [17]. Early weaned Brahman-cross steers kept onsite before transportation were more tolerant to transport stress and feed lot entry and had improved feed to gain ratios [15]. In conclusion, managerial factors such as stabulation, animal density, grouping and transportation, through activation of autonomic nervous system response and up-regulation of the hypothalamus-pituitary-adrenal axis, can affect not only animal welfare but also disease susceptibility, productive and reproductive performances. However, the cellular mechanisms through which this happens are not completely elucidated yet.

#### Nutrition, hormones, and physiological outputs

There are direct connections between nutrition and the endocrine system. Nutritional status and body energy reserves are important to the hypothalamic-hypophysis-gonadal axis integrity in cattle [18]. Many hormones that are influenced by digestive physiology, including secretin, growth hormone (GH), insulin, and insulin-like growth factor I and II (IGF-I and II), carry out important roles in animal reproduction. Thus, idiosyncrasies in nutrition will impact the endocrine systems and vice versa. Leptin is a peptide hormone produced primarily by adipose tissue, and is the putative link between nutritional history, environmental stressors and systems physiology in mammals. Leptin is essential for puberty and postpartum reproduction, and is positively correlated with body condition in ruminants. Interestingly, leptinemia and expression of leptin in tissue are also affected by stage of pregnancy and lactation, colostrum intake, circulating levels of insulin, glucose, glucocorticoids and GH, ingestion of fatty acids, especially linoleic acid, and photoperiod [19]. Dietary restrictions affect the onset of puberty in heifers, weight at calving, mammary growth, milk production and postpartum anestrus in cows [4,20,21]. Environmental estrogens consumed with the diet can act as endocrine disruptors, directly interfering with reproductive function. For example, phytoestrogens and xenoestrogens stimulate endothelin-1(ET-1) synthesis in the oviduct cells. Considering that ET-1regulates tubal contractility, environmental estrogens may have deleterious effects on embryo transportation and implantation [22]. Heifers born from dams receiving protein supplement during gestation tended to be younger at puberty and had a greater pregnancy rate [20].

A critical time period exists during which neuroendocrine functions can positively be manipulated through nutrition. For example, female calves fed a high energy/high protein milk replacer were younger and weighed less at the onset of puberty and two weeks younger at conception and calving than those on a low energy diet [23]. Dietary treatment during early calfhood changes gene expression in higher brain centers, creating a link between feed intake, energy expenditure and reproduction. Heifers fed a high concentrate diet had a decreased gene expression of neuropeptide Y in the arcuate nucleus, subsequently affecting GnRH secretion and negative effects on GH [4,21]. Previously, researchers suggested pushing heifers to reach 60-65% of the adult body weight before the breeding season, through intensive feeding, but recent research decreased the target body weight to 50-55% [24]. High energy diets caused greater circulating concentrations of leptin, insulin and IGF-I in heifer calves and in cows [4,21,25,26]. IGF-I plasmatic concentration has been positively related to follicular growth and to shorter calf-to-conception intervals [25,26]. Liver and adipose tissue IGF-I response to GH administration is lower in cows with negative energy balance, as during early lactation, likely caused by down-regulation of GH receptors [27,28]. IGF-I decreased the blood fatty acid profile during early lactation after the administration of bovine somatotropin [28], which is in contrast to the previous research that adipose tissue stores were minimal as a result of the negative energy balance. Furthermore, protozoal infection in calves has been shown to cause prolonged thyroid deficiency dictated by reduced feed consumption, subsequent to impaired secretion of thyroid hormones and metabolism [29]. Because triiodothyronine (T3) is essential for GH-dependent IGF-I synthesis in the liver, decreased thyroid activity as a consequence of parasitism can have direct repercussions on growth and reproduction. In summary, nutrition mostly affects reproductive functions such as age at puberty, fertility, calving, mammary growth and milk production. In general, a high concentrate/high protein diet is recommended in heifer calves and cows during states of negative energy balance. Further research is needed to better understand the mechanisms by which specific nutrients influence hormones affecting systems physiology. This may also enhance biotechnology both in cattle and other livestock.

#### Climate, hormones, and physiological outputs

There are a number of climate related stressors such as cold, heat, humidity, rain, ice, and wind that can affect the endocrine system and influence the performance of an animal such as the reproductive system and normal estrous cycle of a cow. However, current knowledge available to scientists and producers is mostly based on heat stress research. Heat stress is defined as a point on a temperature-humidity index (THI) above that is considered the thermo-neutral zone, which has adverse effects on the animal’s performance such as on a cow’s reproductive system and normal estrous cycle. In a study to determine factors needed to measure the degree of stress that the environment places on the performance of an animal, it was concluded that patterns could be distinguished using the temperature-humidity index alone: However, the accuracy was vastly improved when wind speed and solar radiation were quantified and added to the equation [30]. For dairy cattle, a THI of 70 is considered the upper echelon of the thermo-neutral zone [31]. Continued exposure to heat stress has several known physiological effects such as an increase in plasma progesterone in open and cycling cows, which results in problems with breeding [32]. Heat stressed pregnant cows also showed reduced concentrations of estrone-sulfate and increased concentrations of progestin indicating that stress had an impact on hormones originating from both the dam and the fetus and which eventually led to lower calf birth weights and subsequently diminished milk yield and lactation performance [5].

Cows stalled in refrigerated barns had serum cortisol concentrations lower than those of cattle housed outside demonstrating that heat stress can impact reproduction by inhibiting the release of LH by the anterior pituitary [33]. White et al. [34] found that cows were in estrus longer in the summer than the winter or, mounted more times per estrus during the winter than in the spring or summer and that the intervals between each mount was longer in the summer, which infests the obvious, that there were fewer mounts throughout the day. Stressors, especially heat stress, can also affect an animals’ feed intake which in turn affects their potential to produce meat and milk. For example, using measurements of feed intake and the effects on differences in endocrine functions as a basis, Holstein cows fed ad libitum under heat stress consumed four pounds less per day compared to their counterparts under thermally comfortable conditions. Lower concentrations of plasma somatotropin and higher concentrations of IGF-II were proposed to be involved in these effects of heat stress on dietary consumption [35]. Exposure of cows to artificially extended photoperiods (16 hours) increased milk yield anywhere from 6 to 13 percent during the coldest parts of the year [36]. Having realized the significant impact of climate on cattle production and product quality, several lines of research have been investigated to discover the best possible alternatives to help alleviate these negative effects. Analyzing different types of remedies for heat stress, Collier et al. [5] concluded that the basis for negating the effects of heat stress is optimizing the possible means of heat transfer from each animal. These alternatives are limited to the four classical thermodynamic factors; convection, conduction, radiation, and evaporation. Alleviation of heat stress by refrigeration or evaporative air conditions has also been shown to increase pregnancy rates without differences in progesterone or estradiol. In the same study, cortisol concentrations were not different in cows except on day 20 of the estrous cycle at which time cortisol concentrations were lower in the cooled cows [37]. In summary, the major and better studied climate stressor in dairy and beef cows is heat. Temperatures above the THI may affect reproductive cycle, feed intake and growth. Although these are well known side effects, bioinformatics should be used in order to understand the specific mechanisms causing these endocrine dysregulations.

## Conclusions

Considering the frequent occurrence of many of the environmental stressors discussed, simply attempting to avoid all of these stress-inducing situations is not an option. Stressors do not activate the HPA axis when the animal does not perceive the conditions as stressful [2]. Therefore, measures must be taken to reduce the negative impacts of and/or to acclimating cattle to these stressors to the greatest possible degree. Proper management practices, nutrition, and optimum climatic conditions are indispensable for homeostasis and optimum productivity in cattle. Innovative research aimed at determining causes and mechanisms of endocrine dysregulations induced by environmental stressors, is necessary. High impact research on nutritional regimens and on the proper means of controlling exposure of an animal to adverse climatic conditions is also required for efficient animal production systems, health, and enhanced product quality.

## Competing interests

Authors declare that they have no competing interests in this study.

## Authors’ contributions

All authors have designed the study together. TLB, LC, GFC, CGH, KM, AMM, and BEV contributed equally for this study. DK, EM and LC have revised the synthesis, and made the final revisions. LC drew the figure. All authors read and approved the final manuscript.
